# Emergent Management of Gastric Variceal Bleed in the Setting of Acute Pancreatitis-Related Sinistral Hypertension With Partial Splenic Embolization: A Series of Two Cases

**DOI:** 10.7759/cureus.29002

**Published:** 2022-09-10

**Authors:** Avinash D Gautam, Sanket ., Ayushi Agarwal, Rajanikant R Yadav

**Affiliations:** 1 Department of Radiodiagnosis, Sanjay Gandhi Postgraduate Institute of Medical Sciences, Lucknow, IND

**Keywords:** endovascular interventions, gastrointestinal bleed, gastric varices, splenic vein thrombosis, necrotising pancreatitis, partial splenic embolization

## Abstract

Sinistral portal hypertension in the setting of acute pancreatitis is a known complication owing to splenic vein thrombosis. It can lead to upper gastrointestinal bleeding due to the development of fundal gastric varices due to the shunting of blood via short gastric veins. However, in the setting of acute pancreatitis, surgical procedures can have high post-operative morbidity. Emergent management of cases with absent gastro-renal shunt can be done by partial splenic arterial embolization, as it is minimally invasive and can provide similar results. Herein, we report a case series of two cases of acute pancreatitis complicated with splenic vein thrombosis and gastric varices, which were managed by partial splenic artery embolization.

## Introduction

Acute necrotizing pancreatitis is usually managed non-surgically as it is associated with high operative morbidity [1]. Splenic vein thrombosis is a known complication in cases of acute pancreatitis which leads to obstruction in the splenic venous outflow. Sinistral portal hypertension refers to increased pressures limited to the splenic side of portal circulation, which results from obstruction to splenic vein flow [2]. This results in shunting of splenic flow into the portal vein via short gastric veins->gastric intramural/submucosal and perigastric collaterals->left gastric vein, in turn leading to gastric varices, especially in the fundal region. These fundal varices are notoriously difficult to manage endoscopically and may be fatal in the absence of timely intervention. They are typically managed via splenectomy. However, in the setting of pancreatitis, surgery is generally difficult, especially in an acute setting [1]. Splenectomy is also associated with increased rates of infection due to the loss of immune function of the spleen [3].

Few authors have reported splenic artery embolization as a potential minimally invasive technique to aid in such situations and control variceal bleeding [4-6].

Here, we describe a series of two cases of pancreatitis that presented with gastric variceal bleeding that was successfully managed by partial splenic embolization (PSE).

## Case presentation

Case 1

**Figure 1:**
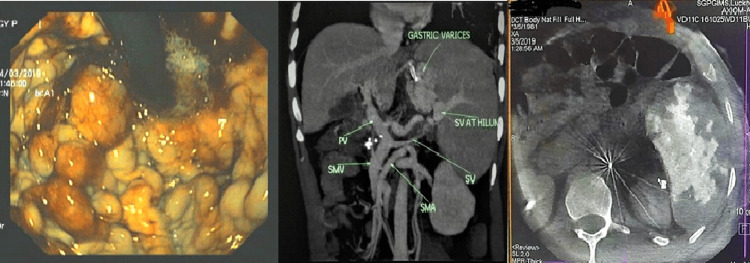
Endoscopic and imaging findings in case 1. The first image is an endoscopic picture showing multiple columns of large gastric varices (>10 mm each) with red-colored signs in the gastric fundus. The middle image is a coronal maximum intensity projection (MIP) CT reconstruction showing attenuated splenic vein (SV) and gastric varices with a patent portal vein. The third image is an intraprocedural cone beam CT, showing patchy enhancement of the spleen post-embolization.

Our index case is a 38-year-old male with alcoholism-related acute chronic pancreatitis that presented with hypovolemic shock secondary to multiple episodes of large volume hematemesis. The patient was initially managed in an outside institute with 12 units of packed red blood cell (PRBC) transfusion over two weeks, with no respite in bleeding and a hemoglobin drop to 2.9 g/dl. Upper GI endoscopy showed large gastric varices. However, endoscopic intervention could not be attempted. A CT study of the abdomen was performed, which showed findings of chronic calcific pancreatitis with attenuated splenic vein and gastric varices in the absence of a gastro-renal shunt (Figure 1).

The patient was referred to our department and was taken up for partial splenic embolization for the management of gastrointestinal bleeding. Under local anesthesia and mild sedation through a transfemoral approach using a 65 cm long 5Fr Cobra catheter (Cook Medical Bloomington, Indiana, USA) and a 130 cm long 2.7Fr microcatheter system (Progreat, Terumo Corporation, Tokyo, Japan), the segmental branches of the splenic artery were selectively cannulated and around 50% of the splenic parenchyma was embolized with polyvinyl alcohol (PVA) particles (300-500 u in size). This led to the cessation of acute bleeding.

Case 2

**Figure 2:**
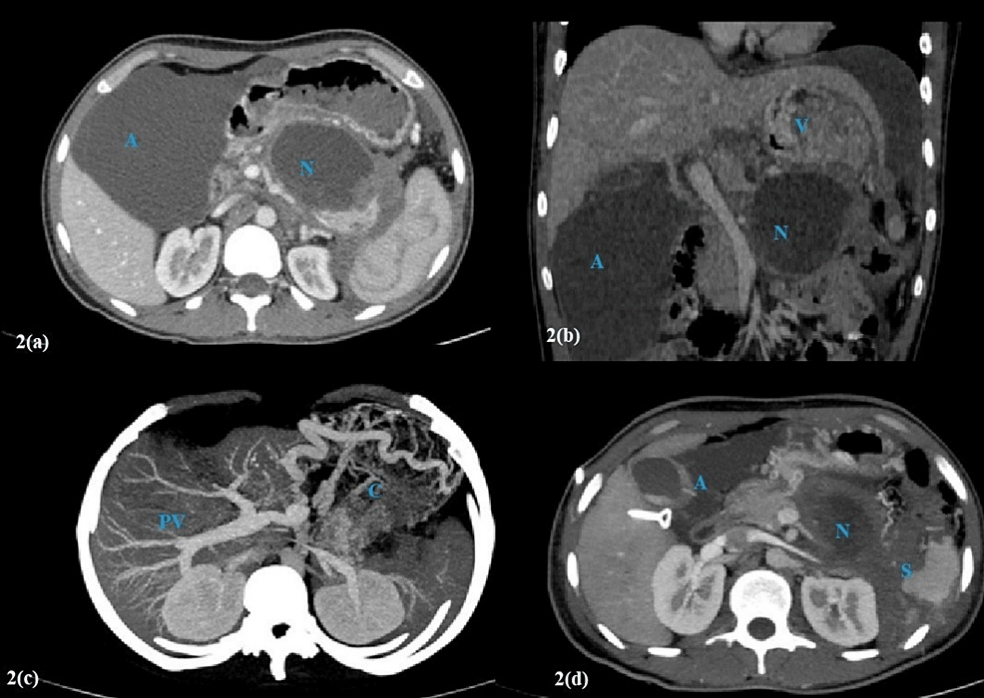
Pre- and post-embolization CECT findings. (a) and (b) are axial and coronal reformatted images showing ascites (A), pancreatic necrosis (N), and varices (V). The portal vein appears normal. (c) is an axial maximum intensity projection image before the procedure showing normal opacification of the portal vein (PV) and its branches and perigastric collaterals (C). Splenic is not visualized. (d) is an axial CECT image, post-partial splenic embolization which shows a partially infarcted spleen (S). Note the reduced lesser sac necrotic collection (N) and ascites (A). CECT: contrast-enhanced computed tomography.

The second case is that of a 24-year-old male with idiopathic moderately severe necrotizing pancreatitis of over three months duration with h/o multiple episodes of gastric variceal bleeding. The CT scan was done, which showed splenic vein thrombosis that was initially managed conservatively (Figure 2). Over a period of three days, the patient had an episodic upper GI bleed with a drop of 2 g/dL in hemoglobin (from 9.4 to 7.4 g/dL), even after two units of PRBC transfusion. Endoscopic glue injection into varices was attempted but was unsuccessful. An emergent splenectomy was considered; however, the patient was found to be at high-risk for surgery in view of comorbidities (necrotizing pancreatitis).

The patient was referred to our department for management of variceal bleed and was taken up for emergent splenic artery embolization, and around 70% of splenic parenchyma was embolized with PVA particles (300-500 u in size). This resulted in the resolution of bleeding.

Post-embolization syndrome was conservatively managed. No fresh episodes of bleeding were noted over three months of follow-up.

Parallelly, the pancreatic pseudocyst was managed by percutaneous trans-gastric drainage with a 10F pigtail catheter (Devon Innovations Private Limited, Bengaluru, India) followed by endoscopic pancreatic duct stenting. On follow-up, a gradual decline in collections and improvement in the patient's symptoms was noted (Figure 2).

## Discussion

Sinistral portal hypertension/left-sided portal hypertension is characterized by increased pressures limited to the splenic circulation with the rest of the portal system being normal. Due to the close anatomic proximity of the splenic vein to the pancreas, common etiologies include pancreatitis, pseudocysts, and pancreatic masses. Other less common causes include prothrombotic conditions, iatrogenic and traumatic injuries, and colonic pathologies [7].

Pathophysiology

Either portal vein thrombosis or tumoral compression/invasion leads to back pressure changes and diversion of splenic outflow via collaterals. The major collateral pathway includes short gastric vein-gastric fundal collaterals-left gastric vein-splenic vein, which leads to the formation of fundal gastric varices.

Diagnosis 

Clinically, the diagnosis is made by classic findings of isolated fundal gastric varices, splenomegaly, and normal liver function tests [7]. However, it is important to differentiate it from generalized portal hypertension due to liver cirrhosis, which is present in many cases of pancreatitis (an overlapping etiology of alcoholism) and can also lead to isolated gastric varices. Contrast CT helps in identifying the cause of left-sided portal hypertension by demonstrating splenic vein thrombosis with collateral channels as described above.

Management

Splenectomy is most generally considered the primary line of management. This is associated with loss of immune function of the spleen, which may lead to overwhelming post-splenectomy infection (OPSI), also known as post-splenectomy sepsis syndrome, with a mortality of up to 50% [8]. Pancreatitis and its complications, along with inflammatory adhesions in the region, make it a high-risk and difficult surgery in many circumstances. Hence, spleen-preserving alternatives are oftentimes more desirable.

According to the American College of Radiology (ACR) appropriateness criteria, partial splenic embolization is an equally good alternative to splenectomy in cases of sinistral portal hypertension [9]. The main advantages of partial splenic embolization are its minimally invasive nature and maintenance of splenic function. This, along with the reduction of hypersplenism and associated thrombocytopenia, provides an added benefit. Splenic vein recanalization and stenting are equally good alternatives when technically feasible, especially when the etiology is that of external compression or chronic pancreatitis, but in cases of ongoing acute pancreatitis, this is not generally appropriate.

Balloon-occluded retrograde trans-venous obliteration (BRTO), a technique generally done in cases of generalized portal hypertension with bleeding gastric varices, has also been described in the setting of sinistral portal hypertension when a gastro-renal shunt is present. Like in our cases, there was no gastro-renal shunt that could have been obliterated via the transfemoral approach. Other techniques like transjugular intrahepatic porto-systemic shunt (TIPS) are not helpful.

Partial splenic embolization

Maddison performed the first splenic artery (total) embolization in a case of cirrhosis in 1973 [10]. Spigos et al. improved on the technique that did the first partial splenic embolization (PSE) in 1979 and demonstrated better outcomes [11]. This technique has been further refined over the years.

Indications

Hypersplenism, certain hemolytic anemias, splenic trauma, cirrhosis with portal hypertension, and sinistral portal hypertension.

Contraindications

Generally, few, the most important being uncorrectable coagulopathy and severe sepsis.

Technique

Two approaches have been described, the more common distal/selective approach, where a microcatheter is used to selectively cannulate and embolize the splenic vascular bed via segmental splenic artery branches. This allows more control over the percentage of the spleen to be embolized but requires more time. In the proximal/non-selective approach, the catheter is generally placed in the splenic artery distal to gastric and pancreatic branches and embolization is done. This allows the procedure to be completed in a shorter duration but with a higher risk of non-target embolization [12]. Various embolic materials have been described: gel foam, polyvinyl alcohol (PVA), gelatin microspheres, alcohol, etc. Polyvinyl alcohol (PVA) particles are the most commonly used agent. Generally, the target for embolization is between 50% and 70% of the splenic parenchyma.

Vaccination

Pre-procedure vaccination against pneumococcal, H-influenza, and meningococcal is employed by some practitioners.

Complications

Many patients develop pain, fever, and vomiting, which is collectively described as a post-embolization syndrome and is considered to be expected sequelae rather than a complication of the procedure. This is generally managed conservatively with antibiotics, analgesics, and antiemetics. Pleural effusion, pneumonia, ascites, and splenic abscess are considered major complications. Splenic abscess is said to be more common in distal embolization, which can be managed with percutaneous drainage and antibiotics [12].

## Conclusions

Sinistral portal hypertension is a potentially life-threatening complication of pancreatitis that can be managed with partial splenic embolization, a safe alternative to splenectomy where available. It has the advantage of being minimally invasive and will preserve splenic immune function. There is a need to develop an awareness of this technique among physicians.
